# Organic Molecules from Biochar Leacheates Have a Positive Effect on Rice Seedling Cold Tolerance

**DOI:** 10.3389/fpls.2017.01624

**Published:** 2017-09-20

**Authors:** Jun Yuan, Jun Meng, Xiao Liang, Yang E, Xu Yang, Wenfu Chen

**Affiliations:** ^1^Agronomy College, Shenyang Agricultural University Shenyang, China; ^2^Liaoning Biochar Engineering and Technology Research Center, Shenyang Agricultural University Shenyang, China

**Keywords:** biochar, cold stress, GC/MS, molecular docking, rice seedlings

## Abstract

Biochar is known to have a number of positive effects on plant ecophysiology. However, limited research has been carried out to date on the effects and mechanisms of biochar on plant ecophysiology under abiotic stresses, especially responses to cold. In this study, we report on a series of experiments on rice seedlings treated with different concentrations of biochar leacheates (between 0 and 10% by weight) under cold stress (10°C). Quantitative real-time PCR (qRT-PCR) and cold-resistant physiological indicator analysis at low temperatures revealed that the cold tolerance of rice seedlings increased after treatment with high concentrations of biochar leacheates (between 3 and 10% by weight). Results also show that the organic molecules in biochar leacheates enhance the cold resistance of plants when other interference factors are excluded. We suggest that the positive influence of biochar on plant cold tolerance is because of surface organic molecules which likely function by entering a plant and interacting with stress-related proteins. Thus, to verify these mechanisms, this study used gas chromatography-mass spectrometry (GC-MS) techniques, identifying 20 organic molecules in biochar extracts using the National Institute of Standards and Technology (NIST) library. Further, to illustrate how these organic molecules work, we utilized the molecular docking software Autodock to show that the organic molecule 6-(Methylthio)hexa-1,5-dien-3-ol from biochar extracts can dock with the stress-related protein zinc-dependent activator protein (ZAP1). 6-(Methylthio)hexa-1,5-dien-3-ol has a similar binding mode with the ligand succinic acid of ZAP1. It can be inferred that the organic molecule identified in this study performs the same function as the ZAP1 ligand, stimulating ZAP1 driving cold-resistant functions, and enhancing plant cold tolerance. We conclude that biochar treatment enhances cold tolerance in rice seedlings via interactions between organic molecules and stress related proteins.

## Introduction

Low temperature is one of the most significant environmental factors that negatively influences crop yields globally (Masoomi-Aladizgeh et al., 2015). Chilling stress can increase the production of reactive oxygen(O) species (ROS), decrease total chlorophyll synthesis, damage plant cell membranes, and reduce enzyme activity (Thomashow, 1999; Wang et al., 2017). Plants utilize a huge variety of complex mechanisms to adapt to low temperature stress, directly sensing and reacting to signals and instituting tolerance responses (Zhang et al., 2016); two indicators of cellular oxidative stress, malonaldehyde (MDA) and hydrogen peroxide (H_2_O_2_), for example, are known to be affected by low temperatures (Hu et al., 2006). Plant cells have a protective system that includes enzymatic antioxidants that combat oxidative stress; three examples, superoxide dismutase (SOD), peroxidase (POD), catalase (CAT) are widely distributed in plants and enable the adaption and regulation of ROS in response to abiotic stresses, especially chilling (Li et al., 2015). Cryoprotective soluble sugars are also found in leaf tissues that enhance tolerance to freezing (Strimbeck et al., 2008; Xu et al., 2015), while the natural amino acid L-α-proline (Pro) stored in cell cytoplasm functions as an osmolyte to resist cold and to protect the structure and function of numerous proteins and enzymes (Fedotova and Dmitrieva, 2016). Plant genomes are also known to contain numerous transcription factors which play important roles in a number of processes including responses to abiotic and biotic stress (Udvardi et al., 2007). A variety of genes induced by low temperature have been identified in rice, including kinase and transcription factor regulatory proteins (Seki et al., 2002; Rabbani et al., 2004; Nakashima et al., 2009); Dubouzet et al. (2003) separated five complementary DNA (cDNA) regions that are dehydration responsive element binding (DREB) homologs in rice (i.e., *OsDREB1A, OsDREB1B, OsDREB1C, OsDREB1D*, and *OsDREB2A*), responsible for low temperature-induced expression of *OsDREB1A* and *OsDREB1B* (Dubouzet et al., 2003). The gene *OsWRKY76* encodes a group of transcription factors within the IIa WRKY family in rice (Yokotani et al., 2013); over-expression of this gene leads to enhanced sensitivity to fungi within the family Magnaportheoryzae as well as improved resistance to cold stress (Yokotani et al., 2013). Similarly, over-expression of the R2R3-type MYB gene *OsMYB2* in rice imparts enhanced tolerance to dehydration, salt, and cold stress (Yang et al., 2012). Experiments show that when the novel cold-induced genes *OsCOIN* and *OsiSAP8* are over-expressed in transgenic rice lines, salt, drought, and chilling tolerance are all significantly enhanced (Liu et al., 2007; Kanneganti and Gupta, 2008).

Biochar is derived from the pyrolysis of biomass at temperatures >250°C either in the absence of O or when supply is very limited and is known to exert a number of effects on plant ecophysiology (Lehmann and Joseph, 2015). For example, in a 3-year field trial, addition of biochar influenced the performance of a grass crop and caused remarkable increases in foliar nitrogen (N) and above-ground biomass (Jones et al., 2011). Biochar can also provide a direct source of plant soil nutrients; its presence can impact root growth, and therefore plant performance (Prendergast-Miller et al., 2013), while its application is known to ameliorate the effects of a range of abiotic and biotic stresses in rice and other plants (Elad et al., 2011; Fahad et al., 2015). One result, for example, has shown that biochar mitigates negative effects on two herbaceous plant species via salt sorption (Thomas et al., 2013), and application is known to preserve rice pollen given high-temperature stress (Fahad et al., 2015). Biochar can also improve the growth and leaf N production of quinoa given a sufficient water supply as well as enhance drought tolerance (Kammann et al., 2011). No reports to date, however, have discussed the influence of biochar on cold-stress responses.

Understanding the nature of the organic molecules present on the surface of biochar has developed into an important research area in recent years. Spokas et al. (2011), for example, performed a qualitative analysis of adsorbed volatile organics present on biochar (Spokas et al., 2011), while Buss and Graham (2015) inferred the content and composition of these compounds (Buss and Graham, 2015). A number of studies have suggested that the organic composition of biochar surfaces might have negative effects on plant growth, including decreases in biomass, leaf area, and plant height (Lievens et al., 2014; Buss and Graham, 2015; Kołtowski and Oleszczuk, 2015; Gale et al., 2016), while others have noted that these chemicals can in fact stimulate plant growth at low doses, even though they are biocidal or phytotoxic at higher concentrations (Graber et al., 2010). One further report has even noted that biochar might have a positive effect on plant growth because of the volatile constituents that remain on its surface (E et al., 2015).

Naturally occurring small organic molecules have always attracted research interest because of their varied biological attributes (Bhuiya et al., 2017). At the same time, proteins perform numerous important roles in organismal biology, including in metabolic activity, catalysis, immune responses, and signal transduction. Many of these processes involve interactions between proteins and small organic molecules, and lead to subsequent changes in the conformation of the former. As a result, these modified proteins can induce positive or negative effects in organisms, while the organic molecules can function by acting as signaling substances, auxin analogs, and active components (Antunes et al., 2011; E et al., 2015; Bhuiya et al., 2017). A number of methods to study these interactions have therefore been developed in recent years, including the bioinformatics tools molecular docking (Skrt et al., 2012; Bhuiya et al., 2017). This tool enables an understanding of the three dimensional (3D) structure of receptor proteins and organic molecular ligands via continuous optimization of the location, conformation, and rotated dihedral angle within molecules, as well as amino acid side chains and skeleton of the receptor. The aim of this method is to determine the lowest energy ligand and receptor conformation when they combine in the active region (Pagadala et al., 2017). More than 60 different docking tools and programs have been developed over the last two decades for both academic and commercial use, including DOCK, AutoDock, and FlexX (Wang et al., 2013).

The focus of this study is rice cold tolerance; because this plant is a very important food crop, its production is closely related to environmental temperature (Challam et al., 2015). We combine the use of experiments and bioinformatics (molecular docking) in this study to determine whether, or not, the addition of biochar has a positive impact on cold stress resistance in rice. The secondary aim of this study is to determine the possible mechanisms that underlie the influence of biochar on rice cold stress tolerance. To do this, we investigated the organic molecules on the surface of biochar and their interactions with stress-regulating proteins to determine how plants produce the necessary physiological and biochemical reactions.

## Materials and methods

### Biochar and leacheates

The biochar for our experiments was generated from fast pyrolysis of rice husks at the Rice Research Institute of Shengyang Agriculture University, China. Rice husks were heated to 400°C at a rate of ~15°C/min before temperature was held constant 1 h. We used biochar generated at 300, 400, and 500°C for pre-experimental steps, but that derived at 400°C for treatments as this leads to the most favorable phenotypes. Then we mixed biochar leacheates with dry soil (Hengyu Company, China) at dose of 0% (control), 1, 3, 5, 7, and 10% (weight/weight), creating different concentrations by washing the corresponding dry weights of biochar with ultrapure water. In order to exclude the effect of cations, anions, and organic matter (OM) present in naturally occurring water, biochar leacheates were then washed with 50 ml ultrapure water, stirring at 25°C for 24 h, and filtered through a 0.22 μm sieve. All bacteria were removed from leacheates and soils by autoclaving at 121°C for 60 min, and all samples were stored at 4°C prior to further analysis.

### Physical and chemical properties of biochar leacheates and soil

This study measured the electrical conductivity (EC) and PH of each different 50 ml concentration of biochar leacheate (Table 1). We also recorded the nutrient contents (i.e., K, Na, Mg, Ca, Cu, Fe, Zn, B, and P) of each sample with an X-ray energy dispersive spectrometer (M410K, BrukerNanoGmbH, Germany), N-NO_3_ and N-NH_4_ contents using a continuous flow analyzer (AA3, SEAL, Germany), and total organic carbon content using an element analyzer (VARIO MACRO CUBE, Elementer, Germany). Soil characterization (Table 2) was carried out at the Elemental Analysis Center of Shengyang Agriculture University, China.

**Table 1:** Physical and chemical properties of biochar leacheates.

**Element**	**Different biochar leacheate concentrations (mg/50 ml)**				
	**1(%)**	**3(%)**	**5(%)**	**7(%)**	**10(%)**
EC (μs/cm)	10.6	11.3	12.1	13.4	14.1
PH (units)	7.23	7.35	7.50	7.82	7.96
K	3.231	3.255	3.317	3.324	3.352
Na	3.163	3.172	3.187	3.189	3.194
Mg	0.022	0.024	0.025	0.031	0.035
Ca	0.036	0.036	0.037	0.039	0.041
Cu	0.000	0.000	0.000	0.000	0.000
Fe	0.000	0.000	0.000	0.000	0.000
Zn	0.000	0.000	0.000	0.000	0.000
B	0.001	0.001	0.001	0.002	0.003
P	0.001	0.001	0.002	0.003	0.005
N-NO_3_	0.0001	0.0003	0.0003	0.0004	0.0006
N-NH_4_	0.0000	0.0000	0.0000	0.0001	0.0002
TOC	45.2	48.5	52.1	56.8	62.7

**Table 2:** Soil characteristics.

**Attribute**	**Value**
Moisture (%)	50%
OM (%)	42%
P (g/kg)	13
N (g/kg)	15
K (g/kg)	12
Si (g/kg)	0.3
PH	6.6
Mg (g/kg)	12

### Plant growth conditions and phenotyping

The Japonica Super Rice Shennong 9816 cultivar were used in this study, sourced from the Rice Research Institute, Shenyang Agricultural University, China. Approximately 120 seeds were germinated in a culture dish in an incubator; germinated seedlings were then sown in small 7 cm diameter pots, 50 ml of different biochar leacheate concentrations were added, and samples were kept in growth chambers for 5 days at 28°C day and night, but subject to a 12 h light and dark cycle at 75% relative humidity. In other to preserve the chemical composition of biochar leacheates, the same pH, and microelements were retained in all pots to mitigate the influence of trace elements on plants. One part of each 5-day-old plant was kept at 10°C all day and night in another growth chamber (subject to the same light-dark cycle and relative humidity) for 3 weeks to simulate cold stress treatment, while the remainder were grown at 28°C for 2 days as normal controls. The experimental temperature and pattern used in this study follow the conditions previously applied by Challam et al. (2015), with treatment time based on when plants developed obvious phenotypes at low temperatures or under control conditions.

Parameters included plant height, dry weight, root length, chlorophyll content, and leaf color for both control and treated plants and used these data to evaluate the effects of low temperature on growth. Chlorophyll was extracted from ground leaves in 80% acetone, absorbance values were measured at 663 and 645 nm, and total chlorophyll contents was calculated following previous work (Porra, 2002).

### Plant physiological characteristics

Plant physiological characteristics (i.e., 0.1 g samples, three repeats for each treatment) were measured at 10°C.

MDA content was measured by using the method described by Meng et al. (2015). Briefly, rice leaves were homogenized in 1 ml of 10% trichloroacetic acid. After centrifugation at 8,000 × g for 10 min at 4°C, 0.2 ml supernatant with 0.6 ml of 0.6% thiobarbituric acid was mixed. This mixture was then heated at 95°C for 30 min, quickly cooled in an ice bath, and was then centrifuged at 10,000 × g for 10 min. Supernatant absorbance was then measured at 532 and 600 nm, respectively, while H_2_O_2_ content was determined following the protocols outlined by Meng et al. (2015). In this step, 0.1 g samples were ground with 0.1% trichloroacetic acid and centrifuged at 10,000 × g for 10 min before absorbance was measured at 390 nm.

Anthrone colorimetry was used to measure soluble sugar content applying the method previously outlined by Song et al. (2011), while the amount of free proline was estimated using ninhydrin colorimetry (Bates et al., 1973; Song et al., 2011).

SOD was measured by using the NBT method outlined by Sun et al. (2015) with some modifications. In this experiment, 0.1 g leaves were ground with 1 ml of extracting solution (i.e., 0.1 M EDTA, 50 mmol/l PBS, 0.1% (v/v) Triton X-100, 2% (w/v) PVP) on ice. The resulting homogenate was then centrifuged at 8,000 × g for 10 min at 4°C, and 0.05 ml of the supernatant was added to the reaction system (i.e., 0.15 ml of 20 μmol/l riboflavin, 0.15 mL of 130 mmol/l Met, 1 ml of 50 mmol/l PBS, 0.15 ml of 750 μmol/l NBT, and 0.15 μl of 0.1 mol/l Na_2_-EDTA). The reaction was initiated by the addition of Met and riboflavin, and system contents were determined using a spectrophotometer at 560 nm. POD was measured following the method proposed by Yu et al. (2015), and CAT was measured using the method described by Li et al. (2015).

### Quantitative real-time PCR (RT-PCR)

Cold-regulated genes were identified in this study using the National Center for Biotechnology Information (NCBI) database (www.ncbi.nlm.nih.gov), while primers for RT-PCR genes were designed and amplified using the Primer 3 online tool (www.primer3). Quantitative RT-PCR (qRT-PCR) was carried out in a 10 μl reaction vessel containing 5 μl 2.5 × RealMaster Mix, 20 × SYBR solution (Tiangen Biotech, Beijing), 0.2 μl of both forward and reverse primers, and 1 μl of diluted cDNA (1:10). PCR amplification was performed using System LightCycler 480 equipment (Roche Applied Science, Germany); our qRT-PCR procedure comprised 95°C for 1 min, followed by 40 cycles of 95°C for 10 s, 55°C for 30 s, and 68°C for 1 min. Values for gene expression were calculated following the method outlined by Ramakers et al. (2003) using delta-delta Ct; all the gene primers used for RT-PCR in this study are listed in Table 3.

**Table 3:** List of primers used in this study.

**Name**	**Sequence (5′–3′)**
*OsDREB1A-F*	CTCGAGCAGAGCAAATACAG
*OsDREB1A-R*	AGTAGTGTCCGTACAGTACC
*OsDREB1B-F*	TCCAAGTCTCCAACCTCAGC
*OsDREB1B-R*	CCCCCAATTTCTGGAGAATC
*OsiSAP8-F*	ATGGAGCACAAGGAGACT
*OsiSAP8-R*	CTAAATTTTGTCAAGTTTCTC
*OsCOIN-F*	ATGAGCTCTCTATGCCCCTTTGCCA
*OsCOIN-R*	CTTGTCATCCAATTGTTT TTGTAGA
*OsMYB2-F*	GGGCTGAAACGCACAGGCAAGA
*OsMYB2-R*	CTGCTTGGCGTGCTTCTGC
*OsWEKY76-F*	CATCGTGCCCGGTGAAGAAGAA
*OsWEKY76-R*	AATTCGGGCAGCTTCTGGAGGAT
*Actin-F*	ACCACAGGTATTGTGTTGGACTC
*Actin-R*	AGAGCATATCCTTCATAGATGGG

### Biochar extraction and GC-MS sample preparation

0.5 g samples of biochar used 100 ml of polar organic solvents as well as the non-polar organic solvents hexane and heptanes in Table 4. In order to evaluate differences in compounds between samples, 100 μl extracts of polar organic solvents were dried by evaporating with N before 50 μl of methoxyamine hydrochloride in pyridine (20 mg/ml) was added as a derivatizating agent; 100 μl of N-methyl-N-(trimethylsilyl) trifluoroacetamide was then added as a second derivatizing agent, and the two mixtures were incubated at 60°C for 45 min. All chemical reagents for this step were purchased from Sinopharm (China) and Sigma Aldrich (USA).

**Table 4:** List of polar and non-polar organic solvents used in this study.

**Polar organic solvent**	**Non-polar organic solvent**
Chloroform	Hexanes
Ethanol	Heptanes
Acetonitrile	
Methanol	
Ethyl acetate	
Dichloromethane	

### GC-MS compound analysis

The GC-MS analyses reported in this study were performed using 7890AGC and 240-MS systems (Agilent, USA), following the procedures outlined by E et al. (2015). Non-polar organic extracts were obtained by applying a temperature program of 100°C for 2 min, increased at a rate of 3°C/min to 260°C held constant for 1 min, before increasing again at a rate of 8°C/min to 320°C held constant for 12 min. Polar organic solvents extracts were then obtained by applying a temperature program of 100°C for 2 min that was increased at a rate of 4°C/min to 260°C for 1 min, before increasing at a rate of 20°C/min to 320°C held constant for 3 min. The GC used in this study was operated in EI mode at 70 eV, scanning across a range between 50 and 650 m/z, with spectra obtained between 50 and 1,000 m/z. We used the NIST08 MS library to identify chromatographic peaks, performing three parallel replicates under the same conditions for each fast pyrolysis.

### Molecular docking analysis

We identified the proteins involved in stress-regulation pathways influenced by organic molecules on the surface of biochar by comparison with the NCBI database, and then utilized the Research Collaboratory for Structural Bioinformatics (RCSB) protein database to obtain their structures. The 3D structures of small organic molecules were established using the software Chamoffice 2010, while specified target proteins and small molecules were determined for docking analysis using the AutoDock tools V.1.5.6 in the software V.4.2 (Scripps Research Institute, USA).

### Statistical analyses

All numerical data were analyzed using the statistical software SPSS (version 17.0) and Microsoft Excel (version 2003). Addition of the suffixes ^*^ and ^**^ signify that different concentrations of biochar exhibit significant differences at the *P* < 0.05 and *P* < 0.01 levels compared to the control.

## Results

### Biochar has a positive impact on rice cold stress resistance

**Figure 1:**
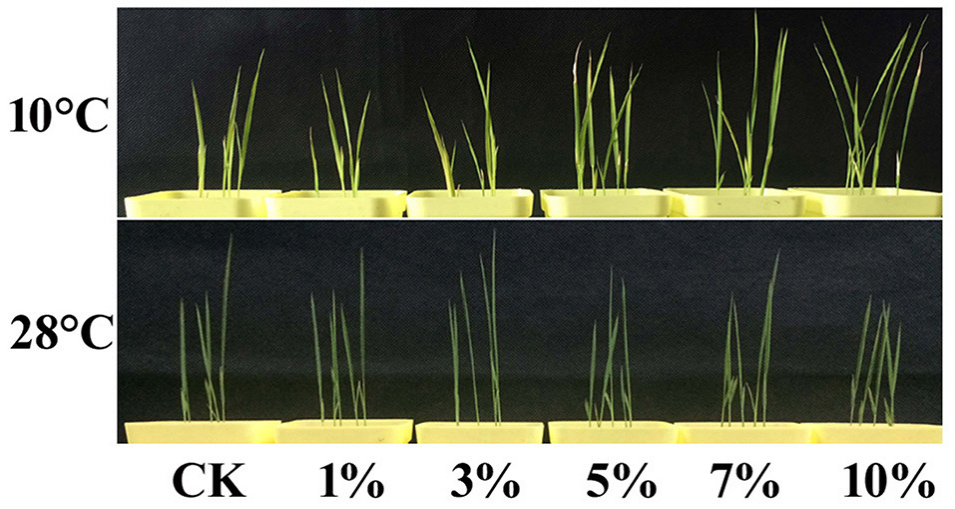
High concentrations of biochar leacheates impart enhanced cold tolerance. Rice plants were subject to different concentrations of biochar leacheates (i.e., control, 1, 3, 5, 7, and 10%). One part of each 5-day-old rice plants was then grown at 10°C for 3 weeks, while non-cold-stressed plants were grown at 28°C for 2 days, following 28°C treatment for 5 days. This figure shows different plant phenotypes.

Rice plants containing different concentrations of biochar leacheates (i.e., control, 1, 3, 5, 7, and 10%) were grown in the same pots under well-watered conditions. Results show that all plants developed the same phenotype when subjected to a temperature of 28°C for 5 days. Thus, to test cold resistance, one part of each 5-day-old rice plant was grown at 10°C for 3 weeks. The phenotypic parameters of all plants are summarized in Table 5. Compared to the control, 1% leacheate treatments reduced plant height by 16.67%, root length by 17.05%, and dry weight by 5.51% (Table 5). Results show that growth of cold-stressed rice plants was worse than that of the control when subject to 1% leacheate treatments (Figure 1; Table 5). However, increasing leacheate concentrations (i.e., 3, 5, 7, and 10%) led to a continuous enhancement in rice plant growth (Figure 1; Table 5); 3, 5, 7, and 10% concentration treatments increased plant height by 12.31, 21.43, 43.37, and 52.84%, respectively, root length by 11.93, 27.27, 43.18, and 52.84%, respectively, and dry weight by 4.24, 8.1, 13.56, 25.42%, respectively (Table 5). At the same time, the control, as well as 1 and 3% leacheate concentrations led to significant yellowing symptoms, while few such symptoms were seen following treatment with 5, 7, and 10% leacheate concentrations (Figure 1). Total chlorophyll contents were therefore measured in order to explain these symptoms; results show that total chlorophyll contents in 1% concentration treatments had decreased by 14.16% compared with the control (Table 5), while in 3, 5, 7, and 10% concentration treatments, total chlorophyll contents increased by 2.6, 16.31, 28.09, and 35.33% (Table 5). Observations show that non-cold-stressed rice plants continued to grow at 28°C for 2 days and that all treatments that comprised an increase in leacheate concentration were characterized by an overall downward trend following an initial increase (Figure 1).

**Table 5:** Phenotypic parameters for one part of each 5-day-old rice plant grown at 10°C for 3 weeks.

**Phenotype**	**Treatment concentrations**					
	**0 (%)**	**1 (%)**	**3 (%)**	**5 (%)**	**7 (%)**	**10 (%)**
Plant height (cm)	10.56 ± 0.89	8.8 ± 0.96^*^	11.86 ± 1.76	13.44 ± 0.59^**^	15.14 ± 1.15^**^	16.14 ± 0.23^**^
Dry weight (mg)	23.6 ± 0.6	22.3 ± 0.5^*^	24.6 ± 0.7	25.5 ± 0.5^**^	26.8 ± 0.4^**^	29.6 ± 0.9^**^
Root length (cm)	1.76 ± 0.13	1.46 ± 0.18^**^	1.97 ± 0.11^**^	2.24 ± 0.05^**^	2.52 ± 0.10^**^	2.69 ± 0.08^**^
Chlorophyll (mg/g FW)	8.83 ± 0.29	7.58 ± 0.04^**^	9.06 ± 0.27	10.27 ± 0.74^**^	11.31 ± 0.41^**^	11.95 ± 0.47^**^

*The error bars in this table are SE, while asterisks indicate statistically significant differences from the control (n = 10*,

* *P < 0.05*,

** *P < 0.01)*.

**Figure 2:**
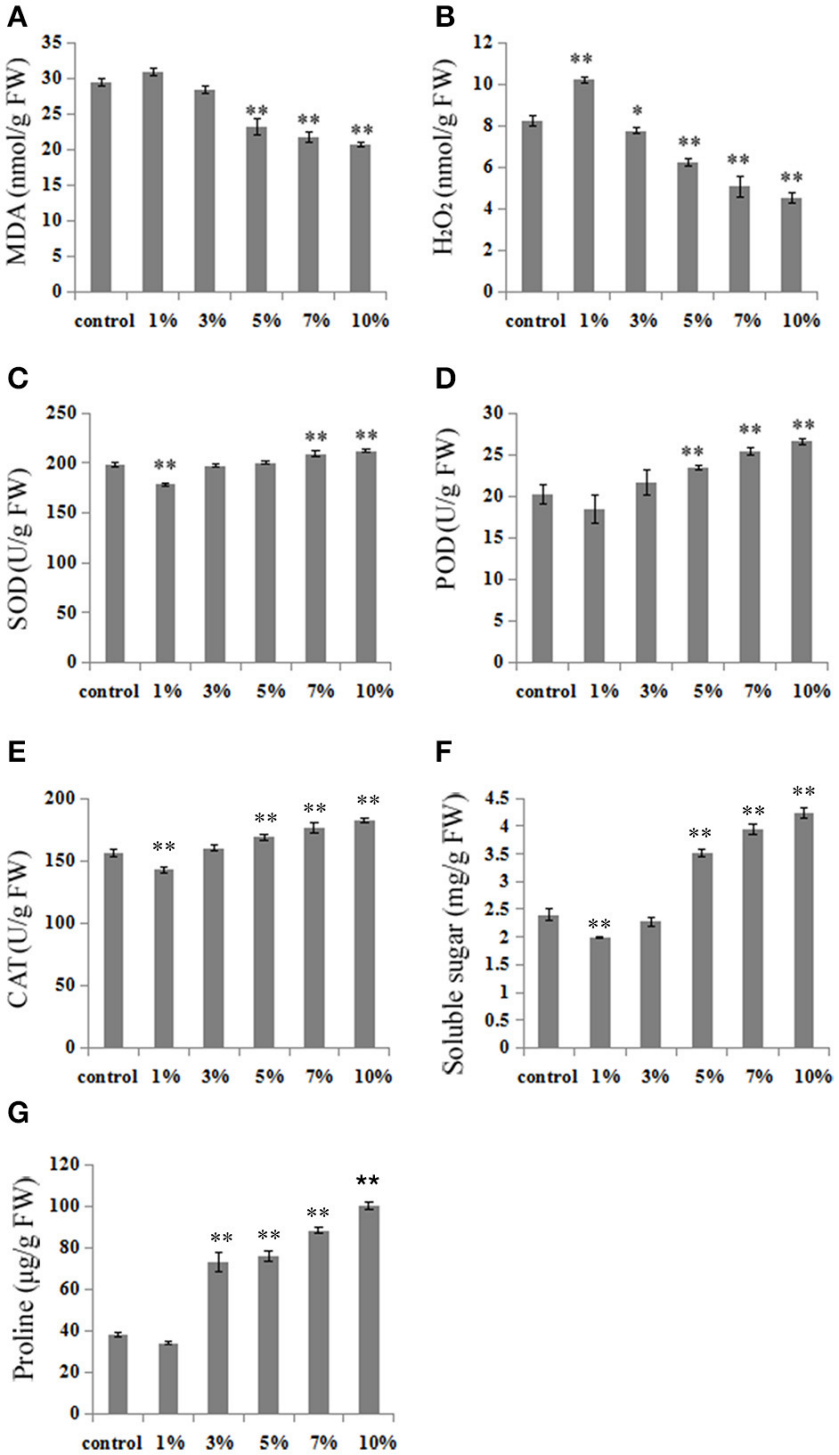
High concentrations of biochar leacheates modulate physiological indexes in response to cold stress. Subsequent to 5 days at 28°C, rice plants were grown at 10°C for 3 weeks. **(A)** MDA concentration, **(B)** H_2_O_2_ concentration, **(C)** SOD activity, **(D)** POD activity, **(E)** CAT activity, **(F)** soluble sugar content, **(G)** proline content. All data are means ± standard error (SE) while asterisks indicate statistically significant differences from the control (*n* = 3, ^*^P < 0.05, ^**^P < 0.01).

Thus, in order to clarify the possible mechanisms leading to cold stress tolerance following treatments with different leacheate concentrations, we analyzed a number of cold stress-related physiological changes, including volumes of MDA, H_2_O_2_, SOD, POD, CAT, proline, and soluble sugars. Results show that subsequent to 3 weeks growth at 10°C, MDA content, a biomarker for cold stress, increased by 5.27% following 1% concentration treatments compared with the control (Figure 2A). At the same time, however, 3, 5, 7, and 10% concentration treatments led to obviously lower MDA contents, respective reductions of 3.5, 21.05, 26.32, and 29.82% (Figure 2A). Results show no difference in tendencies between MDA and H_2_O_2_ contents; compared with the control, the H_2_O_2_ content of 1% concentration treatments increased by 24% (Figure 2B) while the content of this molecule in 3, 5, 7, and 10% concentration treatments deceased by 5.83, 23.94, 38.40, and 45.08%, respectively (Figure 2B).

We also analyzed SOD, POD, and CAT activities, three primary antioxidant enzymes that protect against cold stress in plants. The results of these analyses show that high concentration leacheate treatments (i.e., 7 and 10%) resulted in enhanced SOD activity (increases of 5.4 and 7.1%) compared to the control, in all cases up to levels conducive for cell structure protection (Figure 2C). The activity of POD decreased by 8.71% in 1% concentration treatments, but increased by 6.98, 15.93, 25.28, and 31.47% in high concentration leacheate treatments (i.e., 3, 5, 7, and 10%), compared to the control (Figure 2D). Similar results for CAT activity were seen as for POD; 1% concentration treatments led to a reduction of CAT activity by 8.37% compared to the control, while 3, 5, 7, and 10% concentration treatments led to 3.92, 7.95, 12.86, and 16.59% increases in activity, respectively (Figure 2E).

Plant responses to low temperature are often related to the accumulation of soluble sugars and proline, which can offset resultant osmotic stress. Comparing our control results with those obtained using 5, 7, and 10% concentrations of biochar leacheates reveals a significant increase in soluble sugar contents (46.25, 64.17, and 76.67%, respectively) as treatment proportions increase, while 3, 5, 7, and 10% leacheate concentrations induced significant proline content increases of 91.19, 98.24, 131.5, and 162.6%, respectively. At the same time, 1% concentration leacheate treatments resulted in low accumulations of soluble sugars and proline, decreased of 17.08 and 10.91%, respectively (Figures 2F,G).

**Figure 3:**
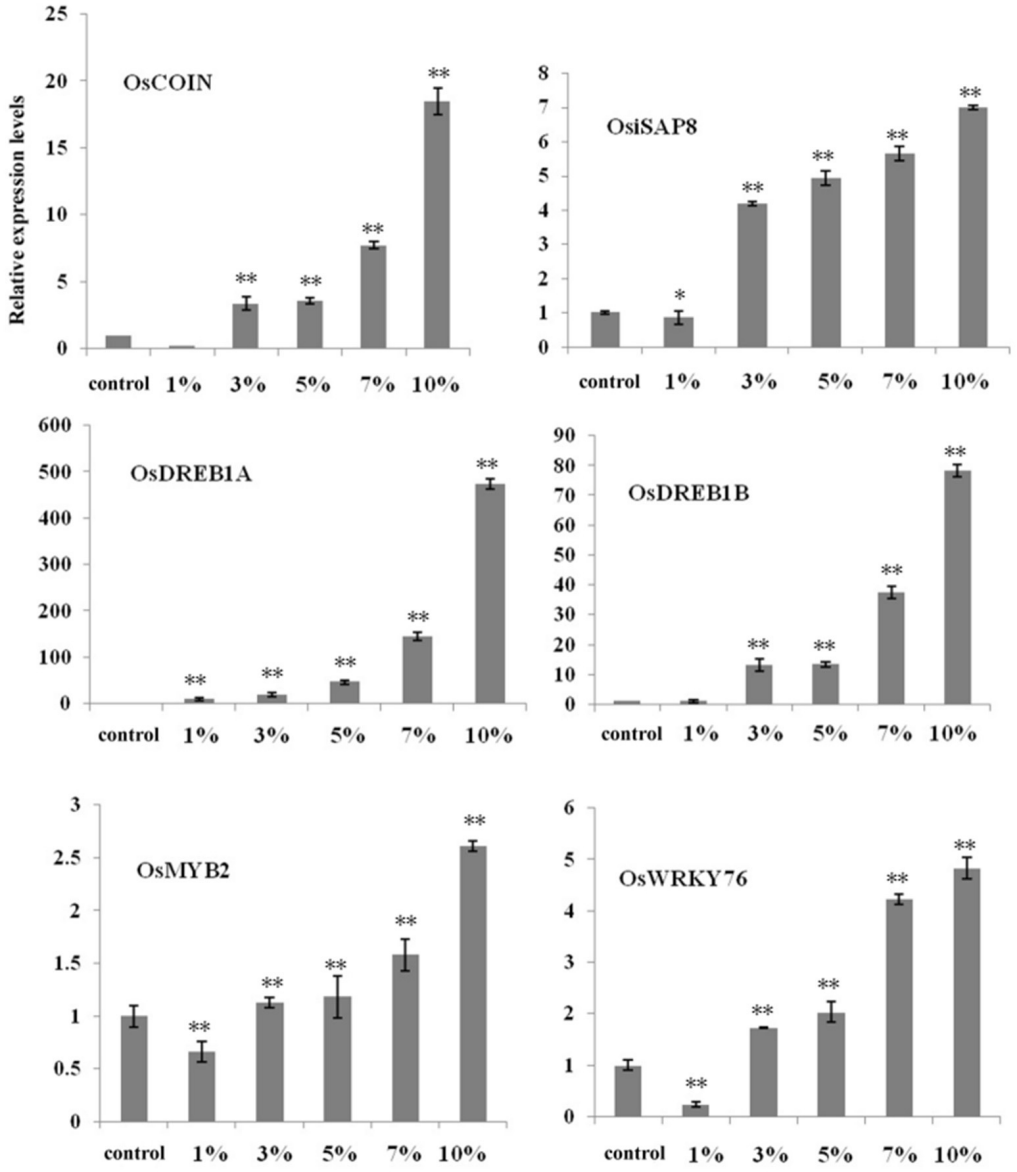
High concentrations of biochar leacheates activate cold response genes in rice seedlings. RT-PCR results performed using cDNA from 5-day-old rice plants grown at 10°C for 3 weeks. Error bars indicate SE (*n* = 3). Asterisks indicate statistically significant differences from control (^*^P < 0.05, ^**^P < 0.01).

To further elucidate the influence of biochar, we selected six important cold-related genes (i.e., *OsDREB1A, OsDREB1B, OsMYB2, OsWRKY76, OsiSAP8*, and *OsCOIN*) known to be involved in the cold signaling pathways. Relative expression analysis using qRT-PCR revealed that the proportion of these transcription factors changed given different biochar leacheate concentrations (i.e., 1, 3, 5, 7, and 10%) compared with the control. Results also show that the expression levels of most genes were down-regulated when a 1% biochar leacheate dose was applied compared with the control, with the exception of *OsDREB1A* (Figure 3). In contrast, increasing biochar leacheate concentrations (i.e., 3, 5, 7, and 10%) led to the up-regulation of all genes (Figure 3), most obviously *OsDREB1A* and *OsDREB1B* (Figure 3). These results suggest that the small organic molecules present on the surface of biochar and involved in improving rice cold tolerance may affect the expression of these transcription factors; cold tolerance improved in plants as biochar leacheate concentration increased. These results show that the addition of biochar has a positive effect on the ability of rice to resist cold stress.

### GC-MS analysis of biochar extracts

**Figure 4:**
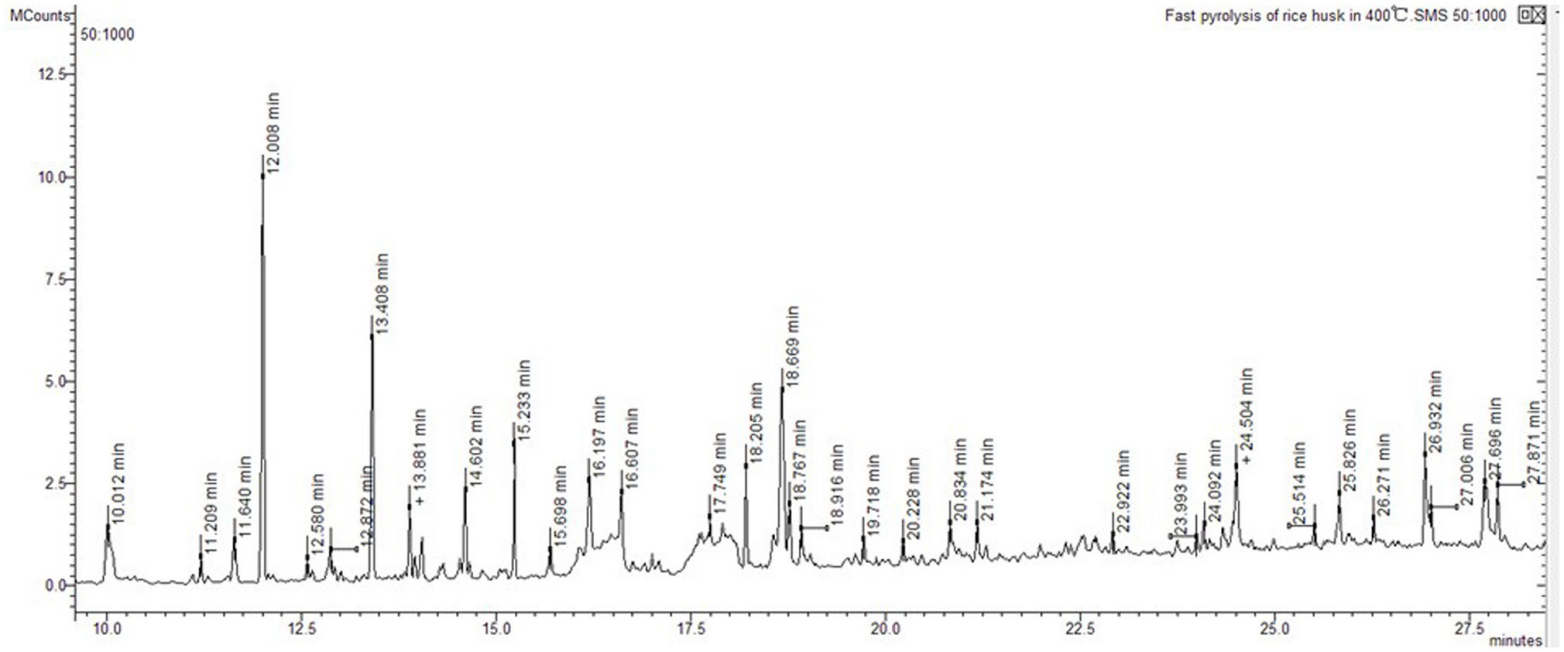
Total ion chromatogram of rice husk fast pyrolysis of at 400°C.

Ion trap MS was employed to analyze pyrolysis products from biochar extracts using polar and non-polar solvents (Figure 4). The electron impact ionization mass spectra we obtained are reproducible and suitable for library matching as comparative collections are readily available. Thus, we used the NIST08 MS library to identify chromatographic peaks; results reveal a total of 20 compounds across the total ion chromatogram, 19 of which were extracted using polar solvents while one was extracted using a non-polar solvent (Table 6). This suggests that numerous polar molecules are attached to the surface of biochar and that many N-containing compounds (more than 50%) occur in different solvents.

**Table 6:** Compounds from biochar extracts identified by GC-MS.

**Solvents**	**Rt (min)**	**Compound**	**Formula**	**Molecular weight daltons**	**Area (%)**
Dichloromethane	2.087	Pyrrole[1,2-a]quinoline-1-ethanol, dodecahydro-6-(2,4-pentadienyl)-, [1R-[1.alpha.,3a.beta.,5a.alpha.,6.alpha.(Z),9a.alpha.]]-	C_19_H_31_NO	289	49.163
	2.260	Pentanamide, 2-(dimethylamino)-4-methyl-N-[2-methyl-1-[[3,3a,11,12,13,14,15,15a-octahydro-12,15-dioxo-13-(phenylmethyl)-5,8-ethenopyrrolo[3,2-b][1,5,8]oxadiazacyclotetradecin-1(2H)-yl]carbonyl]butyl]-	C_36_H_49_N_5_O_5_	631	42.492
	2.755	Pyridine	C_5_H_5_N	79	0.022
	4.523	6-(Methylthio)hexa-1,5-dien-3-ol	C_7_H_12_OS	144	0.118
	5.759	Formamide, N,N-diethyl-	C_5_H_11_NO	101	0.177
	5.925	Ethanamine, N-pentylidene-	C_7_H_15_N	113	0.170
	6.666	trans-2,4-Dimethylthiane, S,S-dioxide	C_7_H_14_O_2_S	162	0.076
	7.022	Acetamide, N,N-diethyl-	C_6_H_13_NO	115	0.111
	7.158	2,2-Diethylacetamide	C_6_H_13_NO	115	0.040
	7.821	Cyclopentanone, 2-(1-methylpropyl)-	C_9_H_16_O	140	0.014
	16.096	2-Acetyl-5-methylfuran	C_7_H_8_O_2_	124	0.018
	18.441	Tritetracontane	C_43_H_88_	604	0.012
Methyl alcohol	2.124	Pentanamide, 2-(dimethylamino)-4-methyl-N-[2-methyl-1-[[3,3a,11,12,13,14,15,15a-octahydro-12,15-dioxo-13-(phenylmethyl)-5,8-ethenopyrrolo[3,2-b][1,5,8]oxadiazacyclotetradecin-1(2H)-yl]carbonyl]butyl]-	C_36_H_49_N_5_O_5_	631	79.126
	2.225	Pyrrolidin-2-one, 1-[1-(4-carbomethoxyphenyl)butan-1-ol-2-yl]-	C_15_H_12_C_l2_O_3_	310	0.0.230
	5.153	6-(Methylthio)hexa-1,5-dien-3-ol	C_7_H_12_OS	144	0.518
	5.741	Ethanamine, N-pentylidene-	C_7_H_15_N	113	0.116
Ethyl alcohol	2.076	(1R,2R,4S)-2-(6-Chloropyridin-3-yl)-7-azabicyclo[2.2.1]heptane	C_11_H_13_ClN_2_	208	1.556
	3.149	6-(Methylthio)hexa-1,5-dien-3-ol	C_7_H_12_OS	144	1.871
	3.938	Formamide, N,N-diethyl-	C_5_H_11_NO	101	1.725
	4.169	1,2-Dimethylaziridine	C_4_H_9_N	71	2.151
	4.892	Acetamide, N,N-diethyl-	C_6_H_13_NO	115	0.912
	5.198	Pyrrole, 2-(4-methyl-5-cis-phenyl-1,3-oxazolidin-2-yl)-	C_14_H_16_N_2_O	228	1.027
Chloroform	2.101	4,6-Dimethyl-2-thioxo-1,2-dihydro-3-pyridinecarbonitrile tbdms	C_14_H_22_N_2_SSi	278	48.326
	2.241	Pentanamide, 2-(dimethylamino)-4-methyl-N-[2-methyl-1-[[3,3a,11,12,13,14,15,15a-octahydro-12,15-dioxo-13-(phenylmethyl)-5,8-ethenopyrrolo[3,2-b][1,5,8]oxadiazacyclotetradecin-1(2H)-yl]carbonyl]butyl]-	C_36_H_49_N_5_O_5_	631	42.529
	4.414	6-(Methylthio)hexa-1,5-dien-3-ol	C_7_H_12_OS	144	0.146
	5.462	Formamide, N,N-diethyl-	C_5_H_11_NO	101	0.213
	5.757	1-Oxa-4-azaspiro[4.5]decan-4-oxyl, 3,3-dimethyl-8-oxo-	C_10_H_16_NO_3_	198	0.191
	7.021	2-Propanamine, N,N-dimethyl-	C_5_H_13_N	87	0.113
Acetonitrile	2.940	6-(Methylthio)hexa-1,5-dien-3-ol	C_7_H_12_OS	144	1.160
	3.650	Formamide, N,N-diethyl-	C_5_H_11_NO	101	1.455
	3.861	Ethanamine, N-pentylidene-	C_7_H_15_N	113	2.480
	4.538	Acetamide, N,N-diethyl-	C_6_H_13_NO	115	1.036
	4.814	2,2-Diethylacetamide	C_6_H_13_NO	115	1.544
	4.948	Cyclopentanone, 2-(1-methylpropyl)-	C_9_H_16_O	140	6.247
Ethyl acetate	2.932	6-(Methylthio)hexa-1,5-dien-3-ol	C_7_H_12_OS	144	2.424
	3.649	Formamide, N,N-diethyl-	C_5_H_11_NO	101	2.005
	3.862	Ethanamine, N-pentylidene-	C_7_H_15_N	113	3.018
	4.529	Acetamide, N,N-diethyl-	C_6_H_13_NO	115	1.089
n-hexane	2.057	Cyclopentane, 1,2,3-trimethyl-	C_8_H_16_	112	100
n-heptane	–	–	–	–	–

### Docking analysis

**Figure 5:**
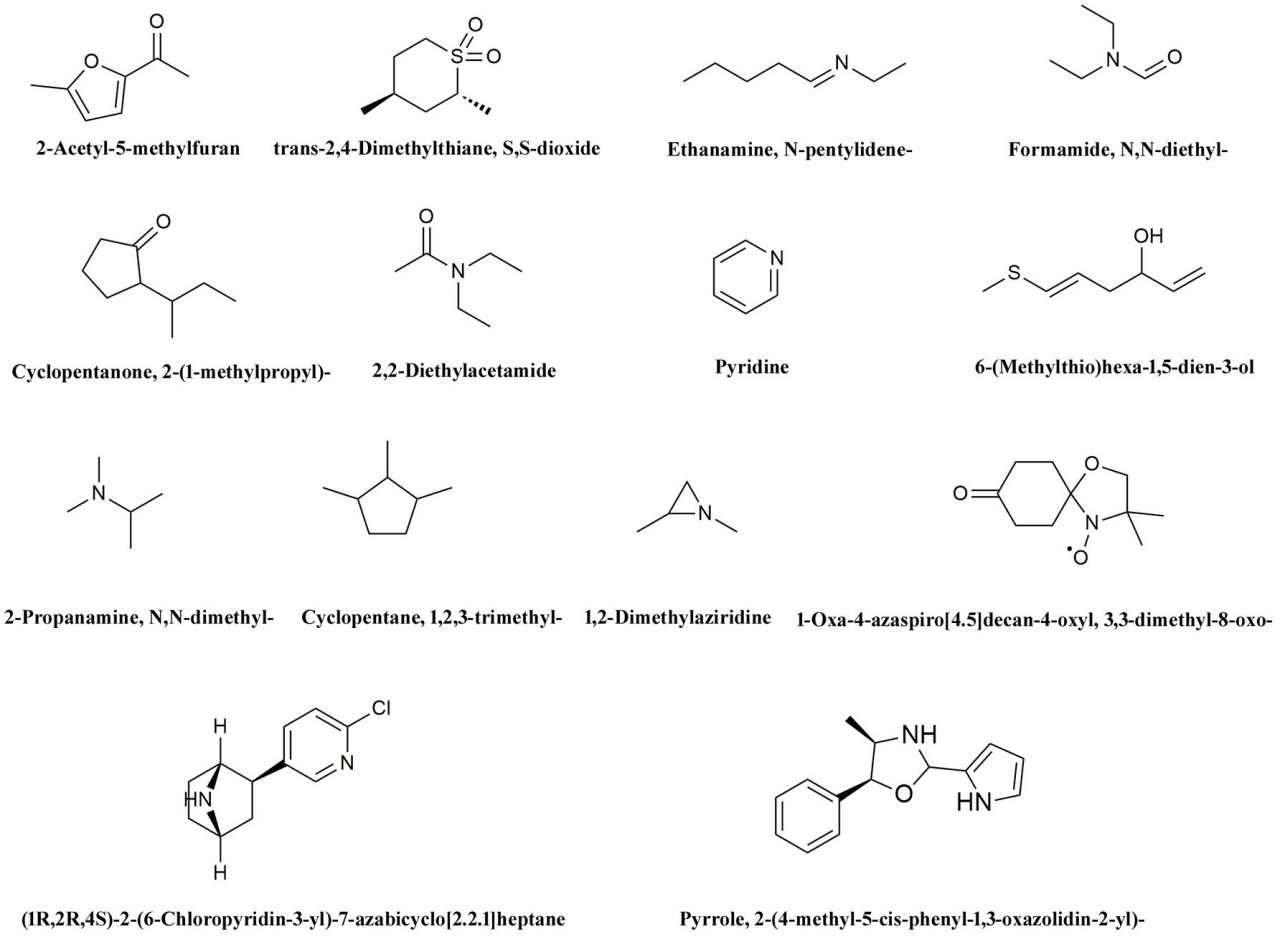
Structural diagrams for 14 candidate organic molecules from biochar extracts.

**Figure 6:**
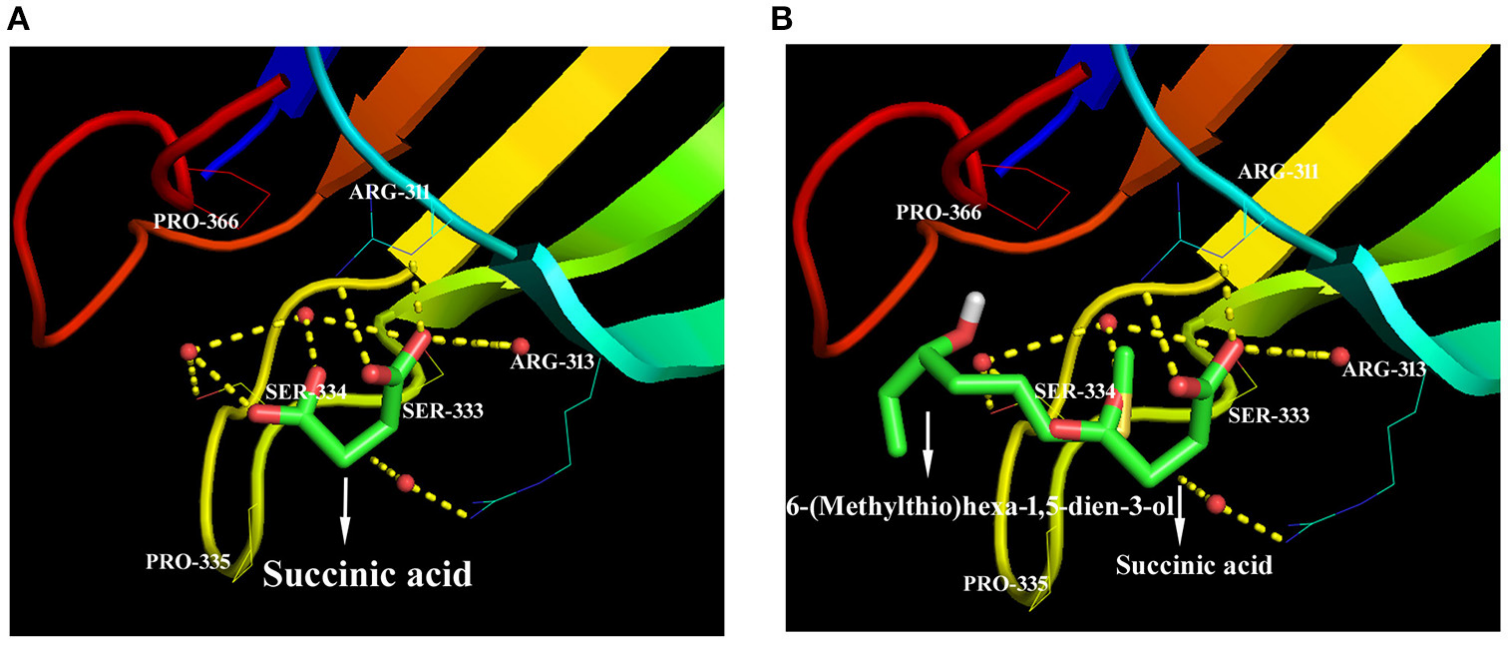
Docked ZAP1 active site with 6-(Methylthio)hexa-1,5-dien-3-ol and succinic acid. **(A)** Succinic acid docked with the ZAP1 active site, **(B)** 6-(Methylthio)hexa-1,5-dien-3-ol and succinic acid docked with ZAP1. These images were drawn using the software PyMOL.

We hypothesize that the organic molecules in biochar that act as ligands are connected to receptor proteins (i.e., stress-related proteins) and thus adjust their functions and influence on plant low temperature resistance. Thus, in order to better understand the mechanisms that underlie the influence of biologically active biochar organic molecules on rice cold stress tolerance, information about binding sites in model proteins is important. We therefore downloaded data from RCSB on the proteins involved in this stress-related pathway, and chose 14 candidate organic molecules which have both low molecular weights and combine easily with proteins and drew diagrams to visualize their structure (Figure 5). Results show that all the candidate organic molecules from biochar extracts acted as ligands for target proteins; for example, succinic acid acts as a ligand for the ZAP1 protein. The binding modes for this acid at active ZAP1 protein sites are shown in Figure 6A, while Figure 6B shows that the biochar organic molecule 6-(Methylthio)hexa-1,5-dien-3-ol is deeply embedded in the active pocket of ZAP1, a favorable binding site. The protein residues in the immediate vicinity of the binding locus for this organic molecule are SER-334, PRO-366, ARG-311, ARG-313, and SER-333. Thus, this molecule along with succinic acid combine with the active site of ZAP1 via a hydrogen bond (Figure 6B); similarly, 6-(Methylthio)hexa-1,5-dien-3-ol mainly interacts with ZAP1 via the formation of these bonds. These results show that the biochar molecular 6-(Methylthio)hexa-1,5-dien-3-ol docks with ZAP1 and acts as a ligand for this protein. This molecule therefore performs the same functions as succinic acid in plants.

## Discussion

Low temperatures have negative effects on rice growth, including the inhibition of chlorophyll biosynthesis. Resultant chlorophyll degradation leads to plant leaf de-greening (yellowing), even the formation of albino leaves (Delmas et al., 2013; Wang et al., 2017). Total chlorophyll contents in the control as well as, 1 and 3% leacheate concentration treatments decreased in this study compared to 5, 7, and 10% treatments; these results suggest that both control and low leacheate concentrations lead to more significant de-greening symptoms than high concentrations. Cold stress also leads to the overproduction of ROS which cause the lipidic peroxidation of plant cell membranes and the production of excess MDA and H_2_O_2_ (Hodgson and Raison, 1991). Thus, MDA and H_2_O_2_ are two important indicators of general lipid peroxidation, a process that results in the destruction of cell membrane structures (Hodges et al., 1999; An et al., 2016; Zhang et al., 2017). Increased MDA and H_2_O_2_ concentrations damage metabolism by causing oxidative injury to rice cells (Zhang et al., 2017); thus, the fact that our results reveal lower MDA and H_2_O_2_ contents following treatment with higher biochar leacheate concentrations at low temperatures (Figures 2A,B) implies that rice plants can alleviate membrane damage when faced with these conditions. Plants have had to develop various methods to mitigate all kinds of biotic and abiotic stresses in order to survive, and SOD, POD, and CAT are all important enzymes from within the antioxidant defense system that mitigate oxidative damage by cleaning up excessive ROS in plants cells under low temperature (Li et al., 2015). Our results also reveal high activity levels of SOD, POD, and CAT following treatment with higher biochar leacheate concentrations under cold stress (Figures 2C–E). These data suggest that high concentrations of biochar leacheates influence SOD, POD, and CAT activity levels and reduce injuries to rice at low temperatures. Proline and soluble sugars are also thought to play important roles as osmoregulatory solutes that improve plant stress tolerance (Delauney and Verma, 1993; Li et al., 2015). The results of this study show that, under cold stress, high biochar leacheate concentrations significantly increased the accumulation of proline and soluble sugars in treatment plants (Figures 2F,G). Data therefore indicate that high concentrations of biochar leacheates closely related to cold tolerance.

Plants respond to environmental stresses by modifying gene expression; in this context, numerous genes have been shown to mediate stress-induced expression (Chen et al., 2014). We extracted six important genes from the NCBI database for evaluation in this study which have been identified as mediators of cold-induced expression. The rice genome contains at least ten *DREB1*-type genes, including *OsDREB1A* and *OsDREB1B*, thought to play key regulatory roles under cold stress (Dubouzet et al., 2003; Challam et al., 2015). In addition, OsCOIN is a RING finger protein which is expressed in all rice organs as the result of cold stress (Liu et al., 2007); over-expression of this protein significantly enhances the levels of some genes known to be cold-responsive and thus the tolerance of rice plants to low temperatures (Liu et al., 2007). Similarly, *OsiSAP8* is a member of the stress associated protein (SAP) gene family (Kanneganti and Gupta, 2008). Over-expression of *OsiSAP8* in transgenic rice results in enhanced tolerance to low temperatures, while chlorophyll retention, fresh weight, root length, and plant height are all greatly enhanced in transgenic rice compared to controls (Kanneganti and Gupta, 2008). It is thought likely that *OsWRKY76* regulates cellular responses to both biotic and abiotic stress (Yokotani et al., 2013), while the over-expression of *OsMYB2* in rice plants also leads to greater up-regulation of cold-related genes and improved tolerance to low temperatures (Yang et al., 2012). The results of this study show that the expression levels of *OsCOIN, OsiSAP8, OsWRKY76, OsMYB2, OsDREB1A*, and *OsDREB1B* were all significantly enhanced given high concentrations of biochar leacheates compared to the control under cold stress (Figure 3). Thus, over-expression of *OsCOIN* significantly enhances cold stress tolerance in rice, accompanied by a concomitant increased in levels of cellular proline (Liu et al., 2007). The expression levels of *OsWRKY76* also exert a positive effect on the expression of lipid metabolism genes (Yokotani et al., 2013), while enhanced SOD activites provide one likely explanation for low H_2_O_2_ contents in *OsMYB2*-over-expressing plants (Yang et al., 2012). These conclusions are corroborated by the results of our physiological experiments (Figure 2). Thus, in sum, high biochar leacheate concentrations cause high expression levels of these genes at low temperatures. These high expression levels also induced a number of cold-responsive genes; these help plants to respond to cold stress while also enhancing protective enzyme activities, proline contents, and eliminating ROS to protect the cell membrane system.

All of these conclusions imply that some biochar leacheate components function to relieve cold stress damage in plants. In this study, we measured and leveled the nutrient elements in biochar leacheates which may influence plant growth and present results which show that the most likely factors leading to enhanced plant cold tolerance are the organic molecules on the surface of biochar. These molecules are washed off in leacheates.

Different feedstocks and types of biochar pyrolysis include different surface organic molecules (Spokas et al., 2011). To date, numerous organic components within biochar have been identified (Spokas et al., 2011); many of these are known to exert positive effects on plants in terms of enhanced height and leaf size (Graber et al., 2010). At the same time, recent research by Gale et al. (2016) has also shown that biochar contains dangerous molecules that inhibit plant growth. Our results nevertheless indicate that, in the absence of other possible factors, the application of high biochar leacheate concentrations (between 3 and 10%) does have a positive effect on rice seedlings at low temperatures. We therefore suggest that the organic molecules present on biochar exert a positive effect on rice growth under cold stress. We used GC-MS to identify 20 organic molecules in biochar extracts in this study (Table 6), a similar result to that reported in previous research (Graber et al., 2010).

This analysis demonstrates that organic molecules on the surface of biochar exert positive effects on the ability of plants to resist cold stress. How these organic molecules function, however, still remains unclear. Research on the interactions between organic molecules and proteins is currently a very active area. In plants, small organic molecules which function in hormonal responses, signaling, and as active substances act as ligands and interact with receptor proteins to trigger rapid biochemical changes and induce intracellular transcriptional and long-term physiological responses (Antunes et al., 2011; E et al., 2015; Bhuiya et al., 2017; Haruta and Sussman, 2017). We therefore speculated that the organic molecules identified in this study might interact with other stress-related proteins, causing positive effects; these interactions thus function to relieve low temperature stresses in plants, as verified in our experiments using molecular docking.

We chose 14 candidate organic molecules for analysis in this study which have low molecular weights and combine easily with proteins (Figure 5). Of these, ZAP1 is member of the WRKY superfamily and is involved in responses to abiotic stress, including drought, cold, and heat (Duan et al., 2007). The molecular docking method requires knowledge of 3D structures of proteins and organic molecules (Wang et al., 2013); we extracted the 3D structure of ZAP1 from the RCSB database and determined the structures of organic molecules using the software Chamoffice 2010. Bioinformatics analysis shows that the compound 6-(Methylthio)hexa-1,5-dien-3-ol in biochar docks with the stress-related protein ZAP1 (Figure 6). However, the functions of 6-(Methylthio)hexa-1,5-dien-3-ol remain unknown, so we are only able to speculate based on the relevant literature. In one comparative case, the abscisic (ABA) ligand interacts with concomitant receptor proteins, resulting in inhibition of type 2C phosphatases and activation of downstream signaling which helps plants to overcome abiotic stresses. Cao et al. (2013) showed that the small organic molecule ABA mimic as well as the original compound have the similar binding mode when they dock with ABA receptor protein and verified that all perform the same biological functions when activiting plant stress-responses. In our study, succinic acid acts as a ligand for the ZAP1 protein as well as a signaling molecule and is indicative of the metabolic state of a plant (Tretter et al., 2016). Thus, 6-(Methylthio)hexa-1,5-dien-3-ol and succinic acid combined with the ZAP1 active site via a hydrogen bond, in other words, they had the similar binding mode of the protein (Figure 6). This suggests that 6-(Methylthio)hexa-1,5-dien-3-ol compound may perform the same functions as the ZAP1 ligand succinic acid. In other words, 6-(Methylthio)hexa-1,5-dien-3-o may act as a signaling molecule that interacts with ZAP1, promoting functioning, participating in some biological activities, and enhancing cold resistance. We did not measure protein expression levels in this study, however, because this would require the use of RNA as a template; if the level of ZAP1 appears to be different at the RNA level, this would demonstrate significant differences at the protein level. Our results do show that expression of the ZAP1 correlation transcription factor *OsWRKY76* did change (Figure 3), indicating corresponding changes in protein levels.

We also analyzed 15, 20, and 30% biochar leacheate treatment concentrations in this study. Results show that these three highest concentration treatments led to similar growth, physiological index values, and related-gene expression proportions as seen after a 10% concentration treatment. These results suggest that, at higher concentrations, the organic molecules from biochar entering plants may already have reached saturation. Thus, the use of 10% biochar leacheate concentrations is the most appropriate and cost effective.

Treatment with a low concentration of biochar leacheates (1%) inhibited the growth of rice seedlings, down-regulated the expression of cold-related genes, and led to a low accumulation of cold-related physiological indexes. Previous research by Thomas et al. (2013) showed that a low dosage of biochar does not mitigate salt stress; the fact that organic molecules in low concentrations trend to have inhibitory affinities with receptor proteins (Lopez et al., 2015) may explain why low concentrations of biochar leacheates (1%) inhibit plants growth.

The results of this study demonstrate that biochar exerts a positive effect on the ability of rice to resist cold stress. We also present new data bearing on the mechanisms underlying the effects of biochar on plant ecophysiology at low temperatures.

## Author contributions

JY designed and performed the experiments, analyzed the results and wrote the manuscript, while JM, YE, XL, XY, and WC designed the experiments.

### Conflict of interest statement

The authors declare that the research was conducted in the absence of any commercial or financial relationships that could be construed as a potential conflict of interest. The reviewer IH and handling Editor declared their shared affiliation.
